# Incidence and associated risk factors of low birth weight babies born in Shaikh Khalifa Bin Zayad Al-Nayan Hospital Muzaffarabad, Azad Jammu and Kashmir

**DOI:** 10.12669/pjms.333.12413

**Published:** 2017

**Authors:** Gulnaz Iltaf, Beenish Shahid, Muhammad Ijaz Khan

**Affiliations:** 1 Gulnaz Iltaf, M.Phil. Department of Zoology, University of Azad Jammu and Kashmir, Muzaffarabad, Pakistan; 2 Beenish Shahid, M.Phil. Department of Zoology, University of Azad Jammu and Kashmir, Muzaffarabad, Pakistan; 3 Dr. Muhammad Ijaz Khan, Ph.D. Livestock Development Research Centre, Muzaffarabad, Azad Jammu and Kashmir

**Keywords:** LBW, Infants, Risk factors, Consanguinity

## Abstract

**Background and Objective:**

Birth weight is the most important factor that affects infant and child mortality. The most common cause of low birth is malnutrition before and during the pregnancy period. The present study was conducted to explore the associated risk factors of low birth weight which will be helpful to undertake effective measures to reduce the incidence of the low birth weight babies.

**Methods:**

The study was conducted at Shaikh Khalifa Bin Zayad Al-Nayan Hospital Muzaffarabad. A sample size of 1603 live births over a period of five months were analyzed. The pregnant women were recruited in the last trimester of their pregnancy and followed up till their delivery. Information regarding maternal age, parity, income of family, gestational age, maternal occupation, degree of illiteracy, birth interval was collected. The birth weight was recorded within 24 hours of delivery. Data analysis was done using Graph Pad Prism version 6.0.

**Results:**

Data of 1863 birth out of which 1603 were live births and among these live births, 1442 were normal birth weight babies and 161 number of low birth weight (LBW) were analyzed. The incidence of LBW in this study was (10.04%). LBW was more common in female (n=84) than in male (n=77) babies. However, this difference was statistically insignificant. Among different risk factors maternal age (p<0.05), parity (P = 0.0167), income of family (P = 0.0190), has a statically significant association with incidence of LBW. The gestational age of mother, maternal occupation, degree of illiteracy was found to affect the incidence of LBW babies, however the difference was found to be statistically insignificant for LBW. Birth interval less than three years and low hemoglobin level (P<0.0260) was found to have a significant association with LBW babies.

**Conclusion:**

LBW a common problem in Pakistan is an important factor for perinatal mortality and morbidity. Among different risk factors maternal age, parity, income of family, gestational age of mother, maternal occupation, degree of illiteracy, birth interval less than three years were found to be the important risk factors contributing to LBW babies born in Shaikh Khalifa Bin Zayad Al- Nayan Hospital Muzaffarabad.

## INTRODUCTION

The babies born with under 2500g weight are term as “low weight” babies. The death rate in low weight new born is forty times compared to normal weight new born.^1^ Annually 15.5% over 20 million infants are born with low weight, this number is 16.5% in less developed and developing countries, 7% in advanced countries and in under developed countries 18.6%. The low birth weight (LBW) has direct relation to renal and cardiac deficiencies, mother’s blood pressure, respiration problems, smoking and drinking alcohol in pregnancy period.^2^ Pre-natal care is very important for both maternal health and fetal development, lack of which have been associated with poor fetal/infant development and LBW babies.^3^ Iron deficiency has been shown to be related with low birth weight and preterm delivery.^4^

Many factors are known to have been associated with LBW babies such as young and old maternal age, multiple gestations, obesity, low socioeconomic status, short stature and previous preterm delivery.^5^ Anemic pregnant women especially in developing countries like Pakistan increase the risk of LBW babies.^6^ Low birth weight babies are thought to have an increased risk of developing type 2 diabetes in later life.^7^ In addition, physical hard work during pregnancy period is one of cause for low birth weight and improper growth.^8^ Interval between two births has significant relation with increasing low weight, death of fetus and prematurity.^9^

In our study the objective was to identify the incidence and the common associated risk factors of low birth weight babies born in Shaikh Khalifa Bin Zayad Al-Nayan Hospital Muzaffarabad.

## METHODS

The present study was conducted in Department of Obstetrics and Gynecology, Shaikh Khalifa Bin Zayad Al-Nayan Hospital, Muzaffarabad, Azad Jammu and Kashmir during the period of February 1, 2013 to June 30, 2013. Mother or close relatives of low birth weight babies were interviewed in labor room and obstetrics ward. The information collected included the birth weight of new born babies, birth order of babies or parity, previous still births and abortions, consanguinity, maternal age, gestational age, birth interval, blood pressure, hemoglobin tests, age at marriage, socio economic status, occupation and maternal education. Any abnormality in mothers like malaria, T.B, eclampsia, diabetes, trauma, hypothyroidism and anemia was collected. Data was collected by using a structured questionnaire. Subjects with incomplete data were excluded. Parity wise birth weight was recorded by using digital scale. At delivery, the gestation age was estimated from ultra-sound data and mother’s menstrual history. Graph Pad Prism version 6.0 was applied for the analysis.

### Ethical approval

This study was approved by the ethical committee of the hospital. All procedures performed during this study and involvements of subjects were in accordance with the ethical standards of the institutional and national research committee and with the 1964 Helsinki declaration and its later amendments.

## RESULTS

Out of total 1863 recruited pregnant women had still born 70 (3.75%) had still bith, abortions accounted for 190 (10.19%) and 1603 (86.04%) were live births. Among these live births, 1442 (89.95%) were normal birth weight babies (NBW) while number of low birth weight (LBW) babies was 161 (10.04%) (Table I). In this study NBW babies were found to had mean birth weight of 3065 ± 12.52g in male and 2791 ± 6.173g in female whereas mean birth weight of LBW babies were 1684 ± 49.0g in male and 1780 ± 48.82g in female.

**Table-I:** Frequency of normal birth weight (NBW), low birth weight (LBW), stillborn and abortions.

*Total No. of Births*	*Live Birth (1603)*		*Stillborn*	*Abortions*

	*NBW*	*LBW*		
1863	1442 (89.95%)	161 (10.04%)	70 (3.75%)	190 (10.19%)

Chi square analysis indicated that gender of the babies did not affect the incidence of LBW or NBW babies (Table II). Different risk factors associated with incidence of LBW babies are presented in Table III. Chi square test for trend showed that a significant decrease in incidence of LBW babies was observed with increase in maternal age. The decrease in number of LBW babies in relation to parity was analyzed by regression analysis of variance which indicated that this decrease was highly significant from zero (P = 0.0167). During the present study, gestational age showed that 21.73% LBW were full term or small-for-dates babies whereas preterm babies were 34.78% in seventh and 43.47% in eighth month of gestation.

**Table-II:** Frequency and gender of normal birth weight (NBW) and low birth weight (NBW)

*Category*	*Male (%)*	*Female (%)*	*Sex Ratio*	*χ^2^_(1)_*	*P*
NBW (1442)	726 (50.34)	716 (49.65)	100:101.39	0.3681	0.54
LBW (161)	77 (47.82)	84 (52.17)	100:91.66		

**Table-III:** Frequency of different risk factors among low birth weight babies (n=161)

*Parameters*	*No. of LBW Babies*	*Percentage of LBW Babies*
***Maternal Age (Years)***		
20-25	78	48.44
26-30	5	36.02
31-35	17	10.55
36-40	8	4.96
***Parity***		
Primigravida	71	44.09
Para 1-3	62	38.50
Para >3	28	17.39
***Gestational Age***		
7 months	56	34.78
8 months	70	43.47
9 months	35	21.73
***Income (PKRs)***		
1,000-10,000	87	54.03
11,000-20,000	46	28.57
21,000-30,000	23	14.2
31,000-40,000	5	3.1
***Maternal Occupation***		
House wife	96	59.6
Working women	39	24.29
Labor class	26	17.39
***Degree of literacy***		
Illiterate	27	16.77
Literate(Primary-Middle)	68	42.23
Matriculation Intermediate	35	21.73
Graduation-Masters	31	19.25
***Birth Interval***		
> 3 Years	33	20.49
< 3 Years	128	79.50
***Patients Hb Level (gm/dl)***		
7.89 ± 0.09	78	48.45
8.55 ± 0.12	58	36.02
8.70 ± 0.30	17	10.55
9.36 ± 0.41	8	4.97

**Fig.1:**
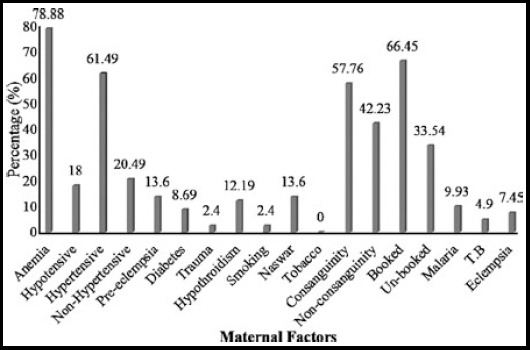
Percentage of LBW babies in relation to various maternal factors.

Most patient with LBW babies (54.03%) were observed in lowest income group whereas only 3.1% were observed in high income group. Regression analysis of variance showed that this decrease in percentage of LBW babies in relation to the income of their parents was significant from zero (P = 0.0190). Proportion of LBW babies was higher (59.6%) in mothers who were house wife while this percentage was lowest 17.39% in mothers belonging to labor class. Degree of literacy of mothers also affected the incidence of LBW as large number of patients (59%) had education less than matriculation including 16.77% illiterate. The birth interval less than three years was noted in 79.50% of patients with LBW. Maternal anemia was seen in LBW patients. Maternal hemoglobin level was found to have a strong association with incidence of LBW babies. Regression analysis revealed that with an increase in hemoglobin level significantly (P<0.0260) There was decrease in the incidence of LBW babies. The number and percentage of LBW babies with different maternal factors is shown in Fig.1. Maternal factors like malaria, tuberculosis, pre-eclampsia, diabetes, trauma, hypothyroidism and smoking and use of Naswar were found to be associated with incidence of LBW babies. Anemia accounted for (78.88%) hypertension (61.49%) and consanguinity (57.76%) which all significantly (P<0.05) increased the incidence of LBW babies.

## DISCUSSION

Incidence of LBW (10.04%) observed in this study is comparable to that observed in the study carried out in in Dow Medical College & Layari General Hospital Karachi where the frequency of LBW was found as 10.6%^10^ and was almost half that of other study (19-23%) in Lahore and Karachi.^11^ In another study done by Jalil, et al. (2016)^12^ the incidence of LBW babies was found to be 24.5% from Punjab. The difference in the incidence of LBW might be due to cultural and racial differences in Lahore, Karachi and other areas of Punjab compared to Muzaffarabad Azad Kashmir. Moreover, the literacy rate of the people is higher in AJ&K. Mean birth weight of LBW babies were significantly lowered than NBW babies in both sexes (P<0.0001). Similar findings were also reported by Kayatha and Tuladhar (2007).^8^ It was observed that maternal health and nutritional status during pregnancy is directly related to the weight of a baby.

In present study, total NBW male and female babieswere found to be 50.34% and 49.65% respectively whereas, total LBW male and female babies were 47.82% and 52.17%. In the current study, highest percentage (48.44%) was observed in maternal age group (20-25years). This is comparable to the study from Horn, et al. (1983); Mondal (1998); Radhakrishan (2000).^13^-^15^ An increase in birth weight was observed in relation to advancing age of mother, this increase is because of increased body stature of the mother with advancement of age. Thus, this study revealed that to get married at an earlier age result in LBW babies, as LBW babies in 1^st^ parity was 71 (32.87%) which was the highest percentage in this study. This finding correlates with study done by Kayastha and Tuladhar (2007),^8^ which were like Moor (1983).^16^

It was observed that in relation to maternal education 16.77% illiteracy was present in LBW mothers. Effect of rate of literacy of the mothers showed statistically significant increase on the birth weight of babies. Similar results were reported by Dhanker (2013), Joshi et al. (2010).^17^,^18^ The highest percentage 54.3% of LBW babies were observed in low income group whereas lowest percentage 3.10% was observed in high income group, this indicated that income of family affect the incidence of LBW babies. Similar results were also reported by Dhankar et al.(2013)^17^, Dickute et al.(2004)^19^ and Joshi et al. (2010).^18^ Thus, educated women with comparatively high income can plan their diet in a good manner during pregnancy which results in good health of their newborn babies.

The pregnancies that terminated at 8^th^ month resulted in higher number of LBW babies (43.47%) compared to the pregnancies that terminated at 9^th^ month (21.73%). Similar results were also reported by Tema (2006)and Siza (2008).^20^,^21^ High incidence of LBW babies was observed in laborer class women compared to house wives. It was in conformity with earlier reports by Viengsakhone (2010), Nobile et al. (2007).^22^,^23^ This indicates that the work stress during pregnancy also affect the birth weight of newborns, this would be avoided as possible.

In present study, the prevalence of LBW babies in anemic mothers was high 78.8% Similar results were also reported by Badshah et al.(2008).^24^ Anemia is a preventable problem and correction of which is expected to result in less incidence of LBW and is likely to lower postnatal mortality in population. In Pakistan, the common cause of anemia during pregnancy is iron deficiency. The supplements of iron during pregnancy is expected to decrease the risk of anemia and to protect the babies against LBW. This was reported by other studies in Pakistan by Khan et al.(2016).^10^ Increased incidence of LBW babies because of consanguinity observed in this study may be due to increased inbreeding because of close marriages among different ethnic groups.

## CONCLUSION

LBW a common problem in Pakistan is an important factor for perinatal mortality and morbidity. Among different risk factors the maternal age, parity 1^st^, month of gestation, low income of family, maternal occupation, illiteracy, short birth interval. Low hemoglobin level and consanguinity were main risk factors found among LBW babies born in Shaikh Khalifa Bin Zayad Al-Nayan Hospital Muzaffarabad, Azad Jammu and Kashmir. The present study recommends that there should be counseling of mothers related to education, not to get marry at an early age, maintaining a birth interval more than three years, supplementation of iron during pregnancy and to avoid the cousin marriages.

### Author’s Contribution

**Gulnaz Iltaf,** did data collection and manuscript writing.

**Beenish Shahid,** worked on concept, designed and supervised the study, review and final approval of manuscript.

**M. Ijaz Khan,** did statistical analysis.
